# Diverse Subsets of γδT Cells and Their Specific Functions Across Liver Diseases

**DOI:** 10.3390/ijms26062778

**Published:** 2025-03-19

**Authors:** Chenjie Zhan, Chunxiu Peng, Huaxiu Wei, Ke Wei, Yangzhi Ou, Zhiyong Zhang

**Affiliations:** 1 State Key Laboratory of Targeting Oncology, National Center for International Research of Bio-Targeting Theranostics, Guangxi Key Laboratory of Bio-Targeting Theranostics, Collaborative Innovation Center for Targeting Tumor Diagnosis and Therapy, Guangxi Talent Highland of Major New Drugs Innovation and Development, Guangxi Medical University, Nanning 530021, China; 202221564@sr.gxmu.edu.cn (C.Z.); 202410267@sr.gxmu.edu.cn (C.P.);; 2 Department of Surgery, Robert-Wood-Johnson Medical School University Hospital, Rutgers University, New Brunswick, NJ 08901-8554, USA

**Keywords:** γδT cells, liver diseases, innate and adaptive immunity, immunotherapy

## Abstract

γδT cells, a distinct group of T lymphocytes, serve as a link between innate and adaptive immune responses. They are pivotal in the pathogenesis of various liver disorders, such as viral hepatitis, nonalcoholic fatty liver disease (NAFLD), alcoholic liver disease (ALD), liver fibrosis, autoimmune liver diseases, and hepatocellular carcinoma (HCC). Despite their importance, the functional diversity and regulatory mechanisms of γδT cells remain incompletely understood. Recent advances in high-throughput single-cell sequencing and spatial transcriptomics have revealed significant heterogeneity among γδT cell subsets, particularly Vδ1^+^ and Vδ2^+^, which exhibit distinct immunological roles. Vδ1^+^ T cells are mainly tissue-resident and contribute to tumor immunity and chronic inflammation, while Vδ2^+^ T cells, predominantly found in peripheral blood, play roles in systemic immune surveillance but may undergo dysfunction in chronic liver diseases. Additionally, γδT17 cells exacerbate inflammation in NAFLD and ALD, whereas IFN-γ-secreting γδT cells contribute to antiviral and antifibrotic responses. These discoveries have laid the foundation for the creation of innovative solutions. γδT cell-based immunotherapeutic approaches, such as adoptive cell transfer, immune checkpoint inhibition, and strategies targeting metabolic pathways. Future research should focus on harnessing γδT cells’ therapeutic potential through targeted interventions, offering promising prospects for precision immunotherapy in liver diseases.

## 1. Introduction

The liver serves not only as the body’s main organ for metabolism and detoxification but also as an essential component of the immune response, continuously surveilling and reacting to pathogens and signals associated with tissue damage [1]. With advancements in immunological research, γδT cells, a unique and multifunctional T cell subset [2], have garnered significant attention for their roles within the hepatic immune microenvironment [3,4]. These cells connect innate immunity and adaptive immunity [5] and have been proved to contribute to infection defense [6], wound healing [7], cancer immunity [8], and transplant rejection [9].

Recent developments in high-throughput single-cell sequencing and spatial transcriptomics have unveiled substantial heterogeneity among γδT cell subsets [10,11,12,13]. Studies indicate that different γδT cell subsets, particularly Vδ1^+^ and Vδ2^+^, exhibit divergent functional properties in liver pathology [14]. For instance, Vδ1^+^ T cells demonstrate potent anti-tumor activity in hepatocellular carcinoma (HCC) by secreting cytotoxic factors and regulating immune checkpoint molecules, thereby inhibiting tumor cell proliferation and metastasis [15]. Conversely, Vδ2^+^ T cells primarily function in inflammation regulation and tissue repair, contributing to immune homeostasis in conditions such as chronic hepatitis B (CHB) and alcoholic liver disease (ALD) [16]. Moreover, research conducted both in vitro and in vivo indicates that γδT cells participate in intricate interactions with macrophages, dendritic cells, and Treg cells, significantly influencing the development and resolution of liver diseases [17].

## 2. Background of γδT Cells

### 2.1. Development and Classification of γδT Cells

**Figure 1:**
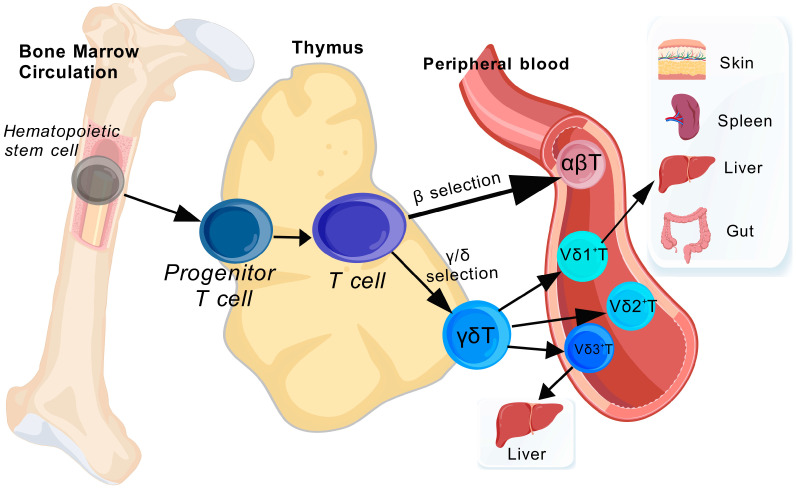
Development and Peripheral Distribution of γδT Cells.

γδT cells, a unique subset within the T cell family, exhibit distinct developmental processes and classification standards compared to conventional αβT cells [18]. γδT cells arise from multipotent hematopoietic stem cells within the bone marrow, subsequently migrating to the thymus, where they undergo a unique differentiation and selection process to reach maturity [19]. Within the thymus, γδT cells undergo rearrangement of the γ and δ receptor chains, generating a diverse set of receptors that offer enhanced specificity and variation in immune responses [20]. In contrast to αβT cells that depend on traditional MHC molecules for antigen presentation, γδT cells have the ability to detect various non-classical antigens, such as stress-related molecules, metabolic byproducts, and lipids. This capability provides them with a distinct advantage in immune monitoring and swift immune responses [21]. While MHC-restricted γδT cells have been observed, they constitute only a minor portion of the overall γδT cell population.To provide a visual overview of their developmental pathway and distribution in peripheral tissues, these processes are depicted in Figure 1 below.

The TCR of γδT cells undergoes a somatic DNA rearrangement process involving the variable (V), diversity (D, exclusive to TRD), and joining (J) regions, generating a wide diversity of individual TCR chain combinations [22]. Furthermore, the combination of TRG and TRD chains, along with the incorporation of palindromic (P) nucleotides and non-template (N) nucleotides by terminal deoxynucleotidyl transferase (TdT) at the V(D)J junction, contributes significantly to increasing the diversity [23]. However, the clonal diversity within an individual’s γδTCR repertoire may be significantly smaller [24].

### 2.2. Classification of Human γδT Cells Based on δTCR Chains

In humans, γδT cells are categorized into three primary subsets—Vδ1^+^, Vδ2^+^, and Vδ3^+^—which are distinguished by the TCR δ chain (encoded by the TRDV gene) [25]. The Vδ2^+^ T cells primarily associate with the Vγ9 chain, creating the Vγ9Vδ2 TCR complex, which represents the most abundant subset of γδT cells in peripheral blood circulation [26]. In contrast to Vδ1^+^ T cells, which develop in the thymus months after birth, Vγ9Vδ2 T cells are generated early during fetal development. This early development indicates their function as crucial components of the body’s natural defense system, acting as the initial line of protection [27]. However, the Vγ9-negative Vδ2^+^ subset exhibits a broader functional diversity, sometimes showing characteristics of adaptive immunity [8].To further illustrate the diversity and functional characteristics of these human γδT cell subsets, their key features are summarized in Table 1 below.

Vδ1^+^ T cells are predominantly located in various tissues, including the intestinal epithelium, skin, spleen, and liver, while only a limited quantity can be detected in peripheral blood [28]. Vδ1^+^ TCRs pair with various Vγ chains, displaying significant flexibility, and their diversity predominantly originates from the TRD gene library rather than the TRG gene library [28]. Sequencing studies indicate that Vδ1^+^ T cells in adults often exhibit a distinct clonal focus, forming private Vδ1^+^ T cell clones [29,30]. These cells can rapidly expand in response to infections or tumors, exhibiting characteristics similar to memory T cells [28,30,31]. This contrasts with Vγ9Vδ2 T cells, which tend to undergo activation-induced cell death (AICD) after prolonged antigen stimulation [32,33]. Furthermore, non-Vγ9Vδ2 subsets, such as Vδ1^+^, Vγ9Vδ2^+^, and Vδ3^+^ cells, can also undergo clonal expansion in both healthy and diseased individuals, leading to the idea that they “record” the immune history of the body, reflecting antigen exposure and immune memory [12]. For example, in infections or tumors [34,35,36,37], the Vδ1^+^/Vδ2^+^ ratio is often inverted in peripheral blood and tissues [38], suggesting that Vδ1^+^ T cells play a crucial role through clonal expansion in chronic infections and tumor microenvironments. Approaches aimed at addressing this issue, such as the adoptive transfer of autologous Vδ2^+^ T cells, could potentially restore the optimal Vδ1^+^/Vδ2^+^ ratio, alleviate tumor burden, and facilitate the reorganization of the tumor microenvironment (TME) [39].

The Vδ3^+^ T cell subset is extremely rare in peripheral blood but is significantly enriched in the liver and intestinal epithelium [40,41,42,43,44]. Its TCR recognizes ligands similar to those recognized by Vδ1^+^ T cells [44] and also expresses high levels of CD16 (a low-affinity IgG Fc receptor, FcγRIII), providing antibody-dependent cellular cytotoxicity (ADCC) capabilities [45]. Vδ3^+^ T cells display increased cytotoxic activity and undergo clonal expansion when infected with malaria, CMV, and HCV [46], highlighting their significant involvement in immune responses to infections. Additionally, recent research demonstrates that Vδ3^+^ T cells infiltrate or proliferate within different tumors, implying a potential function in the immune defense against cancer [47,48,49,50]. In vitro, amplified Vδ3^+^ T cells have been demonstrated to stimulate B cell differentiation and enhance IgM production [51]. However, due to the rarity of Vδ3^+^ T cells, research evidence remains limited, and their functions in physiological and pathological states require further exploration and validation.

This review centers on Vδ1^+^ and Vδ2^+^ T cells, given their extensive representation in existing research and their significance for both experimental studies and clinical applications.

#### 2.2.1. Vδ1^+^ T Cells

Vδ1^+^ T cells play a critical role in adaptive immunity, utilizing γδTCRs and natural killer receptors (NKRs) to facilitate the detection of tumor-associated antigens and stress signals. Although the roles of Vδ1^+^ and Vδ2^+^ T cells in anti-tumor immunity exhibit some overlap, there are notable distinctions between the two subsets. Vδ1 TCRs predominantly identify MHC-like molecules from the CD1 family (e.g., CD1c and CD1d) [52,53,54,55,56,57], as well as membrane-bound proteins such as A2 [58] and MICA/B (MHC class I chain-related proteins A and B) [54,59], which are frequently upregulated in cancerous and virus-infected cells. Studies have shown that the binding affinity of Vδ1 TCRs for CD1d is significantly higher than for MICA/B, suggesting a non-redundant role in specific immune surveillance mechanisms [25]. Similarly to Vδ2^+^ T cells, Vδ1^+^ T cells show increased expression of the stress-responsive molecule NKG2D, which binds to MICA/B present on tumor cell membranes. However, the binding regions for MICA/B vary between the Vδ1^+^ TCR and NKG2D, with the affinity of NKG2D for MICA/B being a thousand times greater than that of the Vδ1^+^ TCR-MICA/B interaction [59].

Although Vδ1^+^ and Vδ2^+^ T cells recognize antigens via different mechanisms, both subsets facilitate the induction of target cell apoptosis through perforin/granzyme B-driven cytotoxic pathways and death receptor signaling (e.g., TRAIL/TRAIL-R and Fas/FasL). These mechanisms highlight that Vδ1^+^ T cells are involved in detecting tumor and stress-related antigens via their specialized TCRs and also triggering cytotoxic responses through various effector pathways. As a result, Vδ1^+^ T cells are essential for the adaptive immune functions performed by γδT cell subsets.

#### 2.2.2. Vδ2^+^ T Cells

The stimulation and antigen recognition of Vδ2^+^ T cells primarily depends on the existence of phosphoantigens. Ligand recognition occurs via two separate pathways: one facilitated by the γδTCR and the other by natural killer receptors (NKR) [60]. In contrast to other γδT cell subsets, the Vγ9Vδ2 TCR primarily identifies phosphoantigens, which accumulate within tumor cells as a result of disturbances in the mevalonate metabolic pathway [61,62,63,64]. However, these phosphoantigens do not directly interact with the γδTCR. Rather, they initially bind to the B30.2 domain of the BTN3A1 protein inside the cell, inducing a conformational alteration in BTN3A1 [65,66]. This, in turn, causes the hinge region of the costimulatory protein BTN2A1 to interact with the Vγ9 chain, ultimately activating Vδ2^+^ T cells [67,68,69,70]. This indirect recognition mechanism has not been fully elucidated, and there remains controversy over whether the Vδ2 chain directly participates in this process. Additionally, the Vγ9Vδ2 TCR can also interact with proteins that are highly expressed in cancer cells, such as F1-ATPase, apolipoprotein A1, and hMSH2, which further enhance its anti-tumor activity [71,72,73]. It is noteworthy that rodents do not possess homologous γδTCRs that can be activated by phosphoantigens, meaning traditional mouse models are unsuitable for studying the capability of Vγ9Vδ2 T cells. Recently, studies in alpacas have revealed Vγ9Vδ2 TCRs responsive to phosphoantigens [74], providing a new non-primate animal model and expanding current research capabilities.

In addition to TCR-mediated recognition, NKR signaling also plays a crucial role in the activation of Vδ2^+^ T cells and the lysis of tumor cells. The NKG2D receptor on the surface of Vδ2^+^ T cells can bind to MICA/B [75,76] and UL16 binding proteins (ULBP) [77,78] expressed by cancer cells, while DNAM-1 interacts with Nectin-like 5, collectively promoting perforin/granzyme-mediated cytotoxic responses [79]. Furthermore, Vδ2^+^ T cells express the CD16 molecule, enabling them to mediate antibody-dependent cellular cytotoxicity (ADCC) through interaction with tumor-specific antibodies [80,81,82]. In vitro studies have shown that this ADCC activity is primarily confined to Vδ2^+^ T cells, whereas Vδ1^+^ T cells generally lack this function [83]. However, CD16 expression has been observed on Vδ1^+^ T cells in patients with viral infections, suggesting functional plasticity across subsets in specific environments [45,84]. Therefore, further investigation into the phenotypic differences in antigen recognition and effector functions between Vδ2^+^ and Vδ1^+^ T cells can help clarify their specific clinical advantages in different pathological contexts, providing new insights for targeted γδT cell immunotherapies.

### 2.3. Effector Subsets Defined by Cytokine Release

The functional properties of γδT cells vary according to the cytokines they secrete, and they can be categorized into distinct subsets based on their effector abilities. Early studies revealed that γδT cells are a primary source of IFN-γ at the onset of immune responses, playing a critical role in anti-tumor immunity across various cancer types [85,86,87,88,89]. However, subsequent research has revealed that γδT cells can, in some contexts, promote tumor growth. For example, in IL-17 knockout mice, γδT cells lacking IL-17 inhibited tumor progression significantly, suggesting that IL-17-producing γδT cells have the potential to promote tumor growth [90,91,92,93,94,95]. Thus, it is difficult to precisely classify the immune functions of γδT cells based solely on TCR chain structures, and researchers tend to classify them into effector subsets based on the types of cytokines they secrete.

The two major effector subsets of γδT cells are γδT1 and γδT17. γδT1 cells secrete IFN-γ and play crucial roles in anti-tumor immunity and intracellular pathogen clearance, while γδT17 cells secrete IL-17, providing protection against extracellular bacterial and fungal infections but potentially contributing to inflammation and disease exacerbation in tumors and certain autoimmune diseases [2,96,97]. Additionally, γδT cells also include a γδNKT subset, which simultaneously secretes IL-4 and IFN-γ, drawing increasing attention [98,99]. The development of these γδNKT and γδT1 subsets relies on strong TCR signaling, whereas γδT17 cells can develop in the absence of TCR ligand selection, highlighting the diverse developmental mechanisms of different subsets [99,100,101,102].

The functionality of γδT cells is highly context-dependent, with their behavior being strongly influenced by the surrounding microenvironment. For instance, γδT1 cells promote anti-tumor immunity within the tumor microenvironment (TME), whereas γδT17 cells, under certain inflammatory conditions, may become dysregulated and contribute to tumor progression [103,104,105,106,107]. Additionally, research has demonstrated that γδT cells exposed to inflammatory cues in the TME can undergo reprogramming into γδTreg cells [103,104,108], which exhibit immunosuppressive functions and attenuate anti-tumor immune responses. This phenomenon is corroborated by the detection of CD73^+^Foxp3^+^Vδ1^+^ cells within the peripheral blood and the tumor microenvironment of breast cancer patients [109]. Similarly, in colon cancer, tumor-infiltrating γδTreg cells frequently display a CD39^+^Foxp3^+^ phenotype, reinforcing their immunosuppressive characteristics. Moreover, CD39^+^ and CD73^+^ γδTreg cells regulate immune responses by modulating extracellular adenosine metabolism, further contributing to immune suppression [110,111]. Studies have also found that under certain pathological conditions, a subset of γδT cells may initiate Th2-like responses by secreting IL-4 [112,113], indicating functional diversity and plasticity. Overall, the characteristics of these effector subsets suggest that the behavior of γδT cells in different tissue microenvironments is highly context-dependent, and their effector functions play important roles in anti-infection, anti-tumor, and immune regulatory processes [40,60,114,115].

Increasing evidence shows that the functions of Vδ1^+^ and Vδ2^+^ T cells are highly plastic and are regulated by the cytokine microenvironment. Typically, Vδ1^+^ T cells are considered to have pro-tumor characteristics, whereas Vδ2^+^ T cells exhibit anti-tumor functions. However, under certain cytokine conditions, the functions of these subsets can shift. IL-6 is a pleiotropic cytokine that is frequently overexpressed in both cancerous and non-cancerous tissues in response to infection and tissue damage [116]. When Vδ2^+^ T cells are exposed to a combination of IL-1β, TGF-β, IL-6, and IL-23, they are capable of differentiating into γδT17 cells that produce IL-17. When exposed to TGF-β1, IL-15, and antigenic stimuli, these cells can be prompted to upregulate FOXP3 expression, resulting in their conversion into Tregs (regulatory T cells) [117,118]. Interestingly, when vitamin C, an epigenetic modifier, is added, the degree of FOXP3 gene demethylation increases, further enhancing its immune regulatory function [118]. Moreover, IL-4 is thought to inhibit γδT cell-driven anti-tumor responses by directing the γδT cell population towards producing the immunosuppressive cytokine IL-10, thereby enhancing the expansion of the Vδ1^+^ subset and suppressing the IFN-γ-producing Vδ2^+^ subset [108]. Clinical studies have shown that in cancer patients, both IL-17-secreting Vδ2^+^ cells and IFN-γ-secreting Vδ1^+^ cells are present [119,120,121], and these two subsets exhibit distinct cytotoxic marker profiles in the tumor environment [122]. Additionally, in the liver, γδT cells are predominantly composed of polyclonal Vδ1^+^ T cells, whose phenotype differs from their counterparts in the blood, suggesting that Vδ1^+^ T cells exhibit greater functional plasticity within the tissue microenvironment [31]. Notably, research by Hayday et al. has associated Vδ1^+^ T cells, rather than Vδ2^+^ T cells, with improved outcomes in individuals diagnosed with triple-negative breast cancer (TNBC), indicating a potential protective function of Vδ1^+^ T cells in certain cancer types [123]. During tumor initiation and progression, γδT cells demonstrate remarkable multifunctionality, with their functional characteristics being highly influenced by tumor type, stage, and microenvironment [8,119]. Furthermore, in addition to their involvement in tumors, γδT cell subsets demonstrate considerable functional diversity and adaptability, allowing them to have either beneficial [123,124,125,126] or harmful [127,128,129] effects in the setting of infections and autoimmune conditions.

### 2.4. Molecular Mechanisms and Signaling Pathways

The activation and functional regulation of γδT cells rely on multiple key molecular interactions, including activating receptors, costimulatory molecules, inhibitory signals, metabolic adaptations, and epigenetic modifications. In contrast to traditional αβ T cells, γδT cells detect antigens independently of MHC restrictions, reacting to stress-induced molecules like MICA/B [130,131]. Among these, the activating receptor NKG2D plays a critical role in triggering cytotoxic responses and promoting the secretion of inflammatory cytokines such as IFN-γ and perforin [59,75]. Additionally, the activation of γδT cells is fine-tuned by costimulatory signals, including CD28 [132] and 4-1BB (CD137) [133]. CD28 sustains γδT cell proliferation and survival by binding to CD80/CD86 while enhancing antigen-specific responses [132]. Meanwhile, 4-1BB and its ligand 4-1BBL enhance the longevity of γδT cells, allowing them to remain functionally active in prolonged immune responses [133].

Although γδT cells possess strong effector capabilities, their activity is tightly controlled to avoid overactivation of the immune system and subsequent tissue injury. The interaction of PD-1 with its ligands, PD-L1/PD-L2, suppresses γδT cell activation, a strategy commonly employed by tumors to evade immune responses [134]. CTLA-4 competes with CD28 for CD80/CD86 binding, thereby suppressing costimulatory signaling and limiting γδT cell activation. LAG-3 interacts with MHC II to further dampen immune responses. These inhibitory signals profoundly impact γδT cells in the tumor microenvironment (TME), contributing to functional exhaustion and diminished anti-tumor immunity [135,136].

The signaling pathways governing γδT cell function involve primarily the NF-κB [137], MAPK [138,139], and PI3K/Akt [140] pathways, which regulate activation, survival, proliferation, metabolic adaptation, and inflammatory responses. However, in TME, abnormal activation of this pathway may promote immunosuppression. Additionally, hypoxic conditions within the TME influence these signaling pathways and induce metabolic reprogramming, ultimately limiting γδT cell persistence and function [141]. Beyond classical signaling pathways, epigenetic modifications significantly shape γδT cell differentiation and function [142,143,144]. Metabolites such as lactate, α-ketoglutarate (α-KG), and acetyl-CoA regulate histone modifications, impacting γδT cell transcriptional programs and determining their activation or tolerance states [145]. While certain epigenetic mechanisms in γδT cells share similarities with αβ T cells, γδT cells exhibit distinct regulatory features in transcription factor dependency and cytokine-mediated modulation. Most studies in this area rely on murine models, and further research is needed to fully elucidate epigenetic regulation in human γδT cells.

Metabolic adaptation is essential for γδT cell function, as these cells predominantly rely on glycolysis rather than oxidative phosphorylation for energy production. This metabolic preference is crucial for sustaining effector functions; however, the hypoxic conditions of the TME limit glycolytic efficiency, thereby compromising γδT cell survival and proliferation [141]. Notably, in glioblastoma models, hypoxia impairs γδT cell anti-tumor activity, while in other cancers, hypoxia enhances cytotoxicity but simultaneously restricts proliferation. Moreover, oxidative stress plays a key role in γδT cell regulation, with reactive oxygen species (ROS) in the TME acting as both inhibitory and modulatory factors [146]. While ROS can suppress γδT cell-mediated immunity, they may also indirectly enhance anti-tumor responses by inhibiting pro-tumor γδT17 subsets [147]. The precise role of ROS in γδT cell function requires further investigation to optimize immunotherapeutic strategies [148].

Exosome-mediated intercellular communication is another crucial factor in γδT cell regulation [149]. Tumor-derived exosomes (TDEs) modulate γδT cell activity via non-coding RNAs such as lncRNA SNHG16, promoting the expansion of immunosuppressive γδT cell subsets like CD73^+^ Vδ1^+^ γδTregs [109]. Furthermore, TDEs may enhance the suppressive function of myeloid-derived suppressor cells (MDSCs), further diminishing γδT cell anti-tumor activity [150]. Conversely, exosomes derived from activated Vδ2^+^ T cells have demonstrated anti-tumor properties and are being explored for adoptive γδT cell-based therapies [151].

Overall, γδT cells demonstrate remarkable functional plasticity, coordinating immune surveillance, tumor immunity, infection defense, and fibrosis regulation through a complex network of activating receptors, inhibitory signals, metabolic adaptations, epigenetic modifications, and intercellular communication. A more comprehensive understanding of the molecular mechanisms of γδT cells will enhance the development of targeted immunotherapies for cancer, infectious diseases, and autoimmune disorders.

## 3. Subset Specific Functions of γδT Cells in Various Liver Diseases

### 3.1. Viral Hepatitis

Viral hepatitis refers to a group of inflammatory liver diseases caused by various hepatitis viruses. Among them, the hepatitis B virus (HBV) and hepatitis C virus (HCV) are associated with high infection rates and significant global disease burden [152,153]. Chronic infections frequently lead to cirrhosis, liver failure, and hepatocellular carcinoma, presenting a significant challenge to public health [152]. With the growing understanding of its pathogenesis, it has become increasingly clear that the host immune response plays a crucial role in regulating virus replication and liver injury, particularly the involvement of γδT cells [154].

The Hepatitis B virus (HBV) represents a major global health concern and is widely distributed around the world. Currently, approximately 257 million people are affected by HBV worldwide, with 68% of cases concentrated in Africa and the Western Pacific region [155]. Among individuals with acute HBV infection, nearly 5% of adults develop chronic hepatitis B, whereas the others clear the virus spontaneously through a self-limiting process [156]. Growing research indicates that variations in HBV infection outcomes are linked to the strength of the antiviral immune response. Several studies have observed a notable rise in the quantity of γδT cells within the liver tissue of acute hepatitis B (AHB) patients, whereas their levels in peripheral blood were found to decrease [157]. Peripheral γδT cells exhibit robust activation, ultimately evolving into memory phenotypes, which enhances their cytotoxic and antiviral functions. In asymptomatic individuals infected with HBV, the proportions of peripheral Vδ1^+^ and Vδ2^+^ T cells are higher compared to healthy individuals, with a significant increase in the levels of IFN-γ^+^ Vδ2^+^ T cells [158]. In the AHB-infected mouse model, a marked expansion of γδT cells in the liver was observed as HBV markers increased. Simultaneously, the expression of activation markers, including CD69, IFN-γ, and IFN-β mRNA, was upregulated [159]. These findings imply that γδT cells may play an antiviral role in AHB patients, potentially restraining the advancement in acute infection.

Following chronic infection, HBV persists by maintaining a stable reservoir of covalently closed circular DNA (cccDNA) within host cells through intracellular genomic recycling and subsequent reinfection, making complete eradication of the virus highly challenging [160]. The role of γδT cells in chronic hepatitis B (CHB) is complex and sometimes contradictory. Several studies have reported a significant reduction in the proportion of Vδ2^+^ T cells in both the liver and peripheral circulation of patients with severe CHB. Additionally, IFN-γ or TNF-α-induced cytotoxicity was compromised but could be reinstated through IFN-α treatment in both in vitro and in vivo conditions [161,162]. It has been reported that, following proliferation in the periphery, human Vδ2^+^ T cells can suppress the release of inflammatory factors from pathogenic Th17 cells, thereby helping to alleviate liver injury [161,163]. However, some studies have indicated that the frequency of γδT cells and their subsets does not significantly differ in CHB patients, with some reports even suggesting enhanced antiviral function in Vδ2^+^ T cells [16]. This implies that variations in patient inclusion criteria (such as age, gender, and ethnicity) may contribute to inconsistent results [164]. In addition, some studies suggest that certain subsets of γδT cells (such as CD4^−^ CD8^−^ types or IL-17-producing cells) may contribute to the persistence and exacerbation of chronic HBV infection by suppressing CD8^−^ T cell activity, promoting the activation of myeloid-derived suppressor cells (MDSCs), or directly enhancing inflammation [165,166]. Combined with the above phenomena, γδT cells often show different functional differentiation states in the process of chronic HBV infection: on the one hand, some γδT cell subsets that can produce IFN-γ or TNF-α have the potential to inhibit the virus and reduce inflammation in the early stage or under specific conditions. On the other hand, some subsets or γδT cells under certain conditions (such as Vδ2^+^ depletion, CD4^−^ CD8^−^, or IL-17 secreting subsets) may aggravate inflammation and immune imbalance, leading to liver injury or disease progression. It is worth mentioning that recent studies have compared the phenotypes of γδT cells in the liver, spleen, thymus and small intestine of mice from embryonic to adult stage, and identified a group of CXCR3^+^CXCR6^+^γδT cells with tissue resident characteristics [167]. It is retained in the liver through chemokines produced by hepatic sinusoidal endothelial cells and generates substantial amounts of IFN-γ during acute HBV infection. The adoptive transfer of these CXCR3^+^CXCR6^+^γδT cells into TCR δ/^−^ mice results in reduced levels of HBsAg and HBeAg, suggesting their protective role in acute HBV infection [167]. It aims to amplify and activate these liver resident CXCR3^+^CXCR6^+^ γδT cells in vivo or in vitro, or it may become a specific immunotherapy for acute HBV infection.

In chronic hepatitis C (CHC), significant attention has been given to the role of γδT cells in pathogenesis. Studies have indicated that the number of γδT cells in the liver of CHC patients is typically higher compared to healthy controls, with Vδ1^+^ T cells predominantly localized in the liver [168,169]. However, compared to healthy controls and asymptomatic HCV carriers, the number of Vγ9Vδ2 and Vδ1^+^ T cells in the peripheral blood of CHC patients tends to be reduced [170]. In the mouse model, compared with the wild type, the level of γδT cells in the liver of HCV transgenic mice was significantly increased [171], suggesting that peripheral γδT cells may be recruited into the liver to participate in the pathological process of HCV infection. Some studies have shown that γδT cells in the liver of CHC patients exhibit high cytotoxic activity and the ability to secrete pro-inflammatory factors [168]. Specifically, IFN-γ^+^ Vδ1^+^ T cells are positively correlated with the extent of liver necrotizing inflammation [169], reflecting the “antiviral-inflammatory” dual effect of γδT cells to some degree. However, even after HCV clearance or antiviral therapy, some patients with CHC still have dysfunction of peripheral γδT cells [46]. It has been observed that although peripheral Vγ9Vδ2^+^ T cells from patients with CHC can express cytotoxic markers such as perforin, granzyme B, and CD107a, their IFN-γ secretion function is significantly impaired [172]. IFN-α intervention can upregulate cytotoxic molecules but cannot restore IFN-γ production ability [173]. Although the new direct-acting antiviral drugs (DAA) treatment can induce slight changes in γδT cell number or TCR spectrum after one year [174], Vγ9Vδ2^+^ T cells in some patients still find it difficult to completely restore their normal cytokine response and proliferation characteristics [46,175]. To some extent, this indicates that the γδT cell functional damage caused by CHC is persistent and can continue to affect the immune balance and liver homeostasis after the virus is cleared.

In conclusion, the function of γδT cells in viral hepatitis is complex and shows obvious heterogeneity: in human acute HBV infection, γδT cells can help inhibit disease progression through enhanced cytotoxic and antiviral activity. In chronic HBV infection, its role is due to subgroup function, inflammatory state, individual differences, and other factors that have protective or pathogenic two-way effects. In CHC, γδT cells in the liver typically exhibit strong cytotoxic activity and pro-inflammatory traits; however, the quantity or functionality of γδT cells in peripheral circulation is frequently impaired, and complete recovery remains challenging even after antiviral treatment. In combination with the above, the Vδ1^+^ subgroup is more likely to reside locally in the liver, participate in direct antiviral and amplify inflammation; the Vδ2^+^ subgroup is mainly active in peripheral blood, which is prone to dysfunction or differentiation deviation in chronic infection. With the continuous advancement in single-cell and multi-group technologies, we will have a clearer understanding of the temporal and spatial distribution, functional diversity, and regulatory network of γδT cells in viral hepatitis and provide more theoretical support for hepatitis prevention and treatment strategies based on γδT cell regulation.

### 3.2. Liver Bacterial and Parasitic Infections

γδT cells are the primary producers of IL-17 during hepatic *Schistosoma japonicum* infection, serving as the initial line of defense before the activation of the T helper 17 (Th17) response. IL-17 is crucial in inducing granulomatous inflammation and fibrosis, both of which can be alleviated by anti-IL-17 monoclonal antibodies [176]. Research has shown that γδT cells producing IL-9 play a role in immune responses in C57BL/6 mice infected with Schistosoma japonicum. In γδT cell-deficient mice infected with Listeria monocytogenes, liver injury is mainly attributed to TNF-α release from CD8^+^ T cells. However, this damage can be reversed by infusing Vδ4^+^ γδT cells that produce IL-10, which in turn modulates CD8^+^ T cell proliferation and decreases TNF-α secretion. However, this pathological damage can be reversed through the infusion of Vδ4^+^ γδT cells that secrete IL-10, which subsequently regulates CD8^+^ T cell proliferation and reduces TNF-α production. Therefore, γδT cells are essential for protecting hepatic tissue by modulating CD8+ T cell responses to pathogenic stimuli and ensuring the maintenance of CD8^+^ T cell homeostasis [177]. Research indicates that γδT cells are particularly significant during the early phase of infection, whereas αβ T cells become more active during the later stages. Moreover, Vδ4^+^ γδT cells’ secretion of IL-17 has been demonstrated to protect against infections. In addition to bacterial and viral pathogens, γδT cells play a critical role in defending against parasitic infections, highlighting their significant immunoregulatory and host-defense functions.

### 3.3. NAFLD and ALD

Recent evidence suggests that alcoholic liver disease (ALD) and non-alcoholic fatty liver disease (NAFLD) share more common mechanisms than previously thought, despite their distinct etiologies. Both conditions are characterized by excessive lipid accumulation in hepatocytes, driven by dysregulated lipid metabolism [178], and exacerbated by oxidative stress from reactive oxygen species [179]. Additionally, they exhibit overlapping inflammatory pathways, including activation of innate immune responses via Kupffer cells and recruitment of pro-inflammatory cytokines (e.g., TNF-α, IL-6), as well as immune dysregulation involving γδT cells and other immune populations [180]. These shared features underscore the potential for common therapeutic targets, such as those explored in γδT cell-based strategies for liver disease. Nonalcoholic fatty liver disease (NAFLD) and alcoholic liver disease (ALD) are two types of metabolic liver diseases, which are characterized by the gradual progress from steatosis to steatohepatitis and even liver fibrosis and cirrhosis [181]. Studies have found that in NAFLD models induced by a high-fat diet (HFD) and high-fat and high-cholesterol diet (hfhcd), γδT cells, especially γδT17 cells produced by IL-17A, are significantly enriched in the liver, and aggravate liver inflammation and injury by recruiting granulocytes and inducing reactive oxygen species [182]. The liver–spleen axis plays a pivotal role in NAFLD by recruiting immune cells, such as monocytes and T cells, to the liver via the portal circulation, potentially serving as a partial source of hepatic γδT cells contributing to inflammation [183]. This immune modulation, alongside cytokine release (e.g., IL-6), amplifies the chronic low-grade inflammation characteristic of NAFLD progression. Moreover, growth factors such as transforming growth factor-β (TGF-β) and platelet-derived growth factor (PDGF) contribute to NAFLD pathogenesis by promoting stellate cell activation and fibrogenesis, further exacerbating liver injury in concert with cytokines and chemokines [184]. Studies have shown that the adoptive transfer of γδT cells into hfhcd-fed mice can accelerate NAFLD, while γδT cell deficiency can protect mice from steatohepatitis, which shows that liver injury and inflammation are significantly reduced [185]. Similarly, CD161^+^γδT cells were enriched in the liver of NAFLD patients, suggesting that γδT cells produced by IL-17A are important regulatory factors for the progression of NAFLD [156].

Besides the IL-17 signaling pathway, γδT cells also affect the progression of NAFLD and ALD through other mechanisms. For example, chronic alcohol intake (ash), methionine choline deficiency diet (NASH), and the combination model of excessive alcohol consumption and high-fat diet showed significant infiltration of γδT cells in the liver, which was regulated by CCR2 [180] and NOD2 [186] signaling pathways. Studies have found that γδT cells are not only the main source of IL-17A but also promote liver injury by regulating the expansion of CD4^+^T cells and inflammatory mode [187]. In addition, γδT cells affect the presentation of lipid antigens through the CD1d-mediated metabolic immune pathway [188], which further activates NKT cells and aggravates liver injuries. It is worth noting that hepatocyte-specific CD1d deletion can improve Nash symptoms in high-fat and high-cholesterol diets [189], which suggests that the CD1d-dependent mechanism plays a key role in the development of steatohepatitis. Akkermansia muciniphila alleviates nonalcoholic steatohepatitis by regulating γδT17 cells and macrophage polarization through TLR2 activation [190].

In conclusion, whether NAFLD or ash, γδT cells produced by IL-17A and CD1d-mediated metabolic immune mechanisms play an important role in the pathogenesis of steatohepatitis. Therefore, targeting these crucial mechanisms, such as blocking IL-17A produced by γδT17 cells or inhibiting CD1d-mediated lipid antigen presentation, may provide a novel approach to treating fatty liver disease.

### 3.4. Liver Fibrosis and Cirrhosis

Liver fibrosis and cirrhosis are the main pathological features of chronic liver disease progression, involving the activation of hepatic stellate cells (HSC) and excessive accumulation of extracellular matrix [191]. Liver cirrhosis is an end-stage disease caused by a variety of chronic diseases. For example, in Asia, hepatitis B virus (HBV) infection is the main cause, while in developed countries, hepatitis C virus (HCV) infection and alcohol abuse are the main factors [191]. The development of liver fibrosis is accompanied by continuous necrotizing inflammatory reaction, which activates immune cells such as HSC and Kupffer cells and ultimately leads to the formation and development of fibrosis [192,193].

In the context of chronic liver injury, γδT cells are one of the important primary immune cells in the portal vein region [194]. These cells promote the activation of HSC and Kupffer cells by secreting interleukin-17 (IL-17), which aggravates the progress of fibrosis [192,195]. Studies have shown that in the *Schistosoma japonicum* infection model, γδT cells recruit centralized granulocytes into the liver through IL-17A, leading to severe liver fibrosis [193]. In addition, the hmgb1-tlr4-il23-il17 signal axis between macrophages and γδT cells plays a key role in the process of liver inflammation [196]. Another study also demonstrated that when IL-23 and IL-1β were present, the binding of exosomes derived from HSCs to TLR3 boosted IL-17A production in γδT cells, which subsequently activated HSCs early on and facilitated the progression of fibrosis [197]. This process highlights the complex interaction between γδT cells and HSCs.

However, γδT cells also showed the opposite effect in the process of liver fibrosis. Some studies have shown that γδT cells induce HSC apoptosis through the expression of Fas ligand (FasL), thereby inhibiting fibrosis in some cases. For example, ccr6^+^γδT17 cells can induce HSC apoptosis in damaged liver tissue through fas/fasl interaction, which helps to limit fibrosis and chronic inflammation [198]. In addition, γδT cells, especially the subsets secreting interferon-γ (IFN-γ), can also enhance their cytotoxic response to HSC by activating natural killer cells (NK cells). This effect depends on the involvement of cytotoxic molecules such as NKp46, trail, and FasL in NK cells, directly or indirectly killing activated HSCs, thus playing a protective role in the process of fibrosis [199].

In general, the role of γδT cells in liver fibrosis and cirrhosis is multifaceted, and its mechanism varies with different cell subsets. γδT cells secreting IL-17A aggravate fibrosis mainly by promoting HSC activation and the production of fibrosis factors, while γδT cells with cytotoxic function play an anti-fibrosis role by inducing HSC apoptosis.

### 3.5. Autoimmune Liver Disease

Autoimmune liver diseases include autoimmune hepatitis (AIH), primary biliary cholangitis (PBC), and primary sclerosing cholangitis (PSC). Their common characteristics are chronic inflammation, immune system imbalance, and autoimmune response to liver or bile duct tissue [200]. The typical manifestations of AIH are periportal hepatitis, elevated serum autoantibodies, and hypergammaglobulinemia [201], while PBC is mainly characterized by the destruction of small intrahepatic bile ducts and the presence of anti-mitochondrial antibodies (AMA) [202]. PSC is characterized by chronic inflammation and fibrosis of bile ducts, which is often associated with inflammatory bowel disease (IBD) [203]. Although the pathological mechanisms of these diseases are different, the potential role of γδT cells in the pathogenesis of these diseases has gradually attracted attention.

Studies have indicated a notable rise in both the proportion and total count of γδT cells in the peripheral circulation, portal venous region, and areas of bile duct proliferation in individuals with autoimmune liver disease, particularly Vδ1^+^T cells [204,205]. The overactivation of Vδ1^+^T cells is considered to play an important role in liver injury and chronic inflammation in AIH and PBC diseases. Flow cytometry analysis showed that the proportion of Vδ1^+^T cells in patients with active PBC was significantly higher than that in the healthy control group and UDCA (ursodeoxycholic acid) treatment full responders, while the level of Vδ1^+^T cells in patients with insufficient responders continued to be high. These cells showed high levels of activation markers HLA-DR, CD69, and CD38 and produced a large number of IFN-γ and TNF-α, suggesting their potential cytotoxicity [205]. In liver tissue, the high expression of Vδ1^+^T cells is closely related to the injury of bile duct epithelial cells and the occurrence of chronic cholangitis. However, the infiltration level of these cells around the interlobular bile duct is low, suggesting that they play a role mainly by circulating into the liver. In addition, the increased release of TNF-α and IFN-γ is considered to be associated with the progression of fibrosis [206]. Thus, the ratio of circulating Vδ1^+^ T cells could serve as a potential marker for monitoring PBC disease activity.

Compared with Vδ1^+^T cells, the content of Vδ2^+^T cells in AIH patients is lower [207]. In addition, γδT cell-derived IL-17A is thought to aggravate hepatocyte injury in the AIH mouse model, and neutralization of the IL-17 pathway can reduce liver inflammation and fibrosis [208]. Vγ4^+^γδT cells showed a protective effect in the ConA-induced liver injury model [192]. IL-17A secreted by Vγ4^+^γδT cells could alleviate liver injury by inhibiting the pathogenicity of NKT cells [4]. However, in the biliary atresia (BA) model, IL-17 released by γδT17 cells is considered to promote the inflammatory response of bile duct and hepatocytes [209], suggesting that γδT17 cells have dual roles in different disease environments.

The immunoregulatory effect of γδT cells also shows that it can induce Treg cell apoptosis and weaken its function in some cases, thereby enhancing the activity of effector T cells and leading to immune imbalance. In addition, γδT cells regulate their activation level by expressing NKG2D receptor [76,210], which is similar to that of NK cells and NKT cells. The study also found that γδT cells can degrade ATP to produce adenosine through extracellular enzymes such as CD39 and CD73 and play an immunosuppressive role, thus inhibiting the hepatitis induced by autoreactive T cells [211]. Therefore, γδT cells can exhibit anti-inflammatory or pro-inflammatory functions under different environmental conditions. The diversity and complexity of their roles make them an important direction in the study of immune regulation of autoimmune liver disease.

In general, γδT cells exhibit considerable functional adaptability and diversity. During the progression of alcoholic liver disease (ALD), various subsets of γδT cells may exert both protective and pathogenic effects on liver tissue. Thus, targeting specific γδT cell subsets, especially through modulation of the IL-17 signaling pathway, presents a promising new strategy for treating autoimmune liver disorders. Although existing studies provide a significant understanding of the role of γδT cells in the development of autoimmune hepatitis (AIH), primary biliary cholangitis (PBC), and primary sclerosing cholangitis (PSC), further extensive and thorough investigations are required to completely clarify the mechanisms involved.

### 3.6. Liver Cancer

The main subsets of γδT cells in the liver are Vδ1^+^ and Vγ9Vδ2. Among them, Vδ1^+^ T cells play a dominant role in the local immune response of the liver, especially in the epithelium and bile duct [212]. In contrast, Vγ9Vδ2 T cells tend to be distributed in peripheral blood and secondary lymphoid tissues [213], and their number is less in healthy liver tissues, but their proportion and functional status will change significantly in liver diseases, such as hepatocellular carcinoma (HCC) and chronic hepatitis [212].

γδT cells show strong anti-tumor ability in the liver tumor immune environment, but its effect function is significantly regulated by the tumor microenvironment (TME) [214]. In a healthy state, γδT cells recognize non-MHC restricted antigens through TCR [215], such as phosphorylated metabolites (such as isopentenyl pyrophosphate, IPP) produced in the metabolic process of tumor cells, and activate rapidly to release cytotoxic molecules such as perforin and granzyme, which can directly kill tumor cells [61,216]. At the same time, these cells enhance the activation of antigen-presenting cells and CD8^+^T cells in the liver by secreting cytokines such as interferon-γ (IFN-γ) and tumor necrosis factor-α (TNF-α), thus forming a broader anti-tumor immune response [217,218]. Studies have demonstrated that γδT cells serve as key effector cells activated following immune checkpoint blockade (ICB) in cancers with HLA class I deficiencies. Their anti-tumor activity is partially mediated through the interaction between NKG2D and its corresponding ligands [219]. However, in the TME of patients with hepatocellular carcinoma, the number of γδT cells, especially Vγ9Vδ2 T cell subsets, decreased significantly, and their effector functions were depleted to varying degrees. High-throughput single-cell analysis revealed that these depleted γδT cells expressed high levels of immune checkpoint molecules, including PD-1, LAG-3 and Tim-3 [220]. In addition, the high expression of LAG-3 is highly correlated with the abnormal glutamine metabolic pathway [220], and the elevated glutamine level is considered to be an important factor in the inhibition of γδT cell function in hepatocellular carcinoma TME [221,222]. Experiments demonstrated that blocking glutamine metabolism could partially recover the functional activity of γδT cells, offering substantial support for the advancement in combination therapies targeting metabolic pathways [220].

Although γδT cells play an important role in the immune defense of liver cancer, they may also be reprogrammed into subsets with immunosuppressive function under certain conditions [214]. In the environment of some tumors and chronic liver diseases, a part of γδT cell subsets will be transformed into regulatory T cells (γδTreg), secreting immunosuppressive cytokines such as IL-10 and TGF-β, thereby inhibiting the activity of effector T cells and NK cells and promoting tumor immune escape [223]. This phenomenon indicates that the functional state of γδT cells is regulated by the cytokine network and metabolic environment in liver TME. In addition, some studies have pointed out that the migration and tissue orientation of liver γδT cells also affect their anti-tumor function [224].CD161 has been shown to promote the migration of Vγ9Vδ2 T cells across the endothelium [225]. Some γδT cells can enhance their homing ability to the liver and tumor sites through the high expression of chemokine receptors CCR5 and CXCR3 [226], but the abnormal expression of chemokines in the immunosuppressive environment may also lead to the reduction in γδT cells’ accumulation in liver tumor tissues [220].

In the study of liver immunotherapy, γδT cells are selectively depleted due to their non-specific recognition of tumor antigen and high killing activity, and cytotoxic Vδ2^+^γδT cells, while Treg-like Vδ1^+^γδT cells are upregulated in HCC. Thus, the infusion of allogeneic Vδ2^+^ γδT cells may offer a promising treatment strategy for this aggressive cancer [96,227,228]. The tumor-targeting properties and efficacy of Vγ9Vδ2 T cells can be markedly enhanced through in vitro expansion [212] or genetic modification of Vδ1^+^ T cells to express chimeric antigen receptors (CAR) [15]. CAR-γδT cell therapy demonstrated significant anti-tumor activity in a liver cancer mouse model. By co-expressing cytokines like IL-15, the survival and persistence of γδT cells in vivo were improved [15]. Additionally, combining γδT cells with PD-1 inhibitors yielded favorable preclinical outcomes, effectively reversing the immunosuppressive environment in the tumor microenvironment (TME) and boosting the anti-tumor activity of γδT cells [229]. However, in order to achieve the wide application of γδT cell immunotherapy in clinical practice, we still need to solve the problems of homing ability, metabolic adaptability, and long-term survival in vivo.

**Figure 2:**
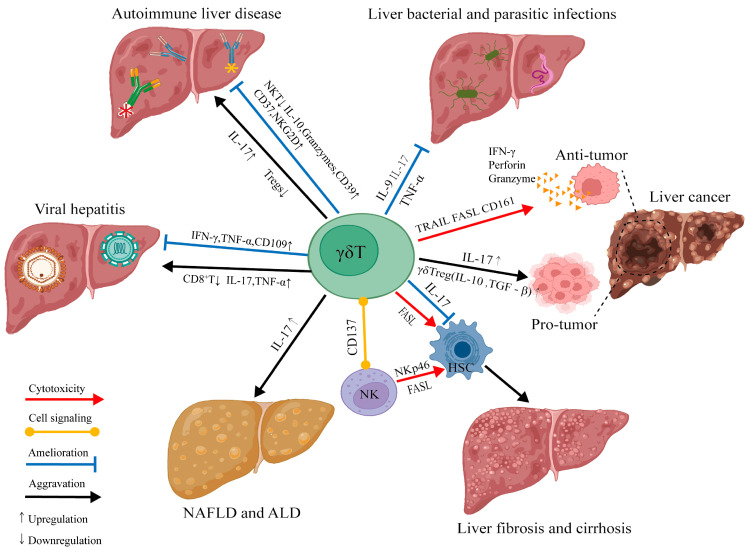
This figure illustrates the dual roles of γδT cells in liver diseases, including viral hepatitis, autoimmune liver disease, NAFLD, ALD, fibrosis, bacterial parasitic infections, and liver cancer. γδTcells exert protective effects by producing IFN-γ, TNF-a, perforin, and granzymes, enhancing immune defense, and regulating immune homeostasis via IL-10 and NKT activation. Conversely, they contribute to fibrosis and tumor progression through IL-17, TNF-a, and FASL, activating hepatic stellate cells (HSCs) and suppressing Tregs. Cytotoxicity is mediated via TRAIL, FASL, and CD16l, while CD137 signaling modulates interactions with NK cells. These insights highlight γδT cells as potential therapeutic targets in liver diseases.

In conclusion, γδT cell subsets play an important role in immune surveillance and anti-tumor defense in the liver. Their functional status in the tumor microenvironment is affected by many factors, such as metabolic regulation, immune checkpoint expression, and cytokine network. Further research on these key regulatory mechanisms will help to provide more accurate targeting strategies for the immunotherapy of liver diseases and liver cancer and provide a theoretical basis for optimizing γδT cell adoptive cell therapy. To visually summarize the diverse roles and interactions of γδT cells in liver cancer and other liver diseases, these mechanisms are illustrated in Figure 2 below.

## 4. Crosstalk of γδT Cells with Immune and Non-Immune Cells in the Liver Microenvironment

γδT cells not only play a “bridge” role between innate and adaptive immunity through their unique TCR recognition and functional differentiation in liver immune regulation [5] but also form a complex crosstalk network with other key immune cell populations. Firstly, during the polarization and activation of liver-specific macrophages (Kupffer cells), γδT cells can promote or inhibit M1/m2 polarization by secreting cytokines such as IFN-γ, IL-17, TNF-α, or directly interacting with Kupffer cell surface ligands and costimulatory molecules [230]. Some studies have shown that when γδT cells tend to express IFN-γ and TNF-α, they can stimulate Kupffer cells to transform into M1 type, amplify the inflammatory response, and mediate some liver tissue damage [231]. However, in another state, some γδT cells (such as IL-10 secreting or immunomodulatory subsets) can guide Kupffer cells to M2 polarization and then play a protective role in the repair of chronic hepatitis or fibrosis [232]. This two-way regulation of “pro-inflammatory and anti-inflammatory” is closely related to the heterogeneity and activation threshold of γδT cells and also reflects the importance of equilibrium in the liver immune microenvironment.

**Figure 3:**
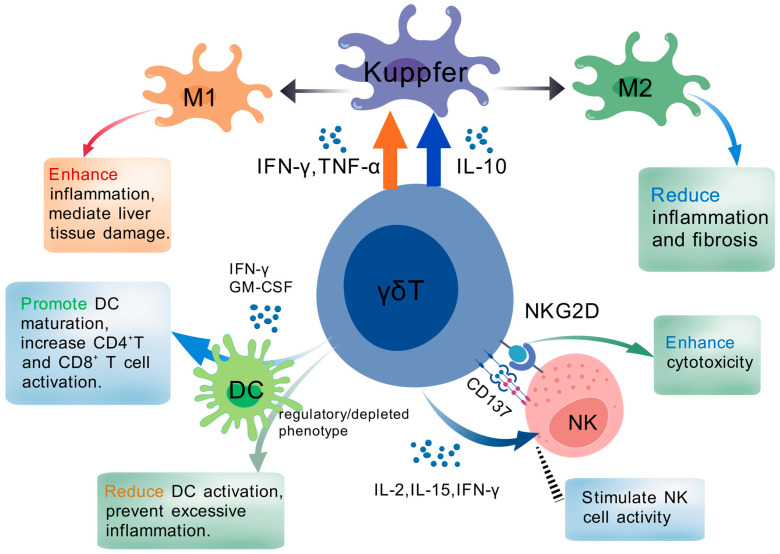
Crosstalk between γδT cells and other immune cells in the liver microenvironment.

At the same time, γδT cells can also interact with dendritic cells (DCS) and natural killer cells (NK cells) synergistically or antagonistically. DCs are responsible for antigen uptake and processing in the early stage of liver immune response [233]. γδT cells can not only transmit pre-excitation signals to DCS, thereby enhancing the presentation efficiency of the latter for intrahepatic pathogens or tumor-related antigens but also enable antigen-like presentation function (γδTapc) under certain conditions [234]. In addition, γδT cells secreting cytokines, such as IFN-γ or GM-CSF, often promote the maturation of DCS and CD4^+^/CD8^+^T cell response [150]. On the other hand, if γδT cells differentiate into regulatory or “depletion” phenotypes, they may reduce the activation intensity of DCS and inhibit excessive inflammation [110]. The collaboration between γδT cells and NK cells primarily governs target cell identification and cytotoxic responses. γδT cells express natural killer receptors (NKRs) such as NKG2D [235], which form signaling circuits with corresponding receptors on NK cells, allowing mutual activation through soluble factors (e.g., IL-2, IL-15, IFN-γ) or costimulatory molecules, thereby enhancing cytotoxicity in a coordinated manner [236,237]. Additionally, γδT cells promote the antifibrotic capacity of conventional NK (cNK) cells and liver-resident NK (lrNK) cells by enhancing their cytotoxicity against activated hepatic stellate cells (HSCs) [199]. This crosstalk between γδT and NK cells is partially dependent on the engagement of the costimulatory receptor 4-1BB (CD137) [133,238]. To visually illustrate the intricate interactions and regulatory effects of γδT cells with Kupffer cells, DCs, and NK cells in the liver microenvironment, these relationships are depicted in Figure 3 below.

Beyond immune interactions, γδT cells engage dynamically with non-immune cells, including hepatocytes, hepatic stellate cells (HSCs), and liver sinusoidal endothelial cells (LSECs), to maintain hepatic homeostasis and influence disease progression [199]. Hepatocytes express stress-induced ligands like MHC class I-related chain A/B (MICA/B) under damage, infection, or oncogenic stress [159], which are recognized by γδT cell NKG2D receptors, triggering cytotoxic responses via perforin and granzyme, and pro-inflammatory cytokine release (e.g., IFN-γ, TNF-α). While this clears damaged cells, excessive activation may exacerbate tissue injury [3]. HSCs, key drivers of fibrogenesis, upregulate ligands such as butyrophilin-like molecules upon activation, engaging γδ TCRs and recruiting γδT cells [199]. In response, γδT cells secrete IL-17 and IFN-γ, which can promote HSC activation and fibrosis or exert immunomodulatory effects to aid resolution, depending on the context [207,230]. LSECs regulate immune trafficking and sinusoidal homeostasis via adhesion molecules (e.g., ICAM-1) and CD1d-mediated lipid antigen presentation [182], inducing γδT cell IL-17 production to amplify immunity or IL-22 to support hepatocyte repair [9]. Collectively, these interactions position γδT cells as pivotal mediators of the liver’s immunological and structural balance, with dual protective and pathogenic roles in conditions like steatohepatitis, fibrosis, and hepatocellular carcinoma [239].

## 5. Clinical Applications, Therapeutic Potential, and Future Directions

In the field of tumor immunotherapy, γδT cells, particularly the Vδ1^+^ and Vδ2^+^ subpopulations, have demonstrated significant therapeutic potential. Although these two subsets share certain chemokine receptors on their surface, such as CCR5 and CXCR1, they exhibit distinct chemokine response patterns and migration behaviors within the tumor microenvironment (TME). For instance, Vδ2^+^ T cells primarily migrate through CCR5-mediated chemotaxis, whereas Vδ1^+^ T cells predominantly rely on CXCR1 and CXCR3, leading to differentiated tissue migration and tumor infiltration patterns [240,241]. Understanding these distinct chemotactic behaviors across various tumor types is crucial for optimizing γδT cell-based immunotherapies. Furthermore, immunosuppressive factors in the TME may reprogram the chemotactic pathways of these cells, redirecting them toward a pro-tumoral migration pattern. Thus, developing strategies to enhance γδT cell accumulation and persistence within tumors remains a critical challenge in improving the efficacy of γδT cell-based immunotherapy.

For allogeneic Vδ1^+^ or Vδ2^+^ T cell immunotherapy, a major challenge lies in extending the persistence of these cells in vivo while maintaining their functional potency. Clinical studies have shown that Vδ2^+^ T cell counts decline sharply within approximately two weeks post-infusion, indicating rapid apoptosis and functional exhaustion within the TME. To sustain the anti-tumor immune response, researchers recommend periodic adoptive transfer therapy (ACT), particularly with multiple infusions before complete tumor remission [39]. Moreover, due to their potent cytotoxicity and antigen-presenting capabilities, Vδ2^+^ T cells may play a pivotal role in post-surgical immune reconstitution in cancer patients.

To overcome the issue of functional exhaustion in the TME, engineered γδT cells have emerged as a promising solution. Through genetic modifications and cellular engineering, researchers can enhance the anti-tumor potency of these cells and extend their persistence within the TME. Among these approaches, chimeric antigen receptor (CAR)-γδT cells enable specific tumor targeting, while genetic modifications to increase resistance to chemotherapy drugs further enhance the efficacy of γδT cells in combination therapies [242,243,244,245]. However, similar to conventional CAR T-cell therapies, CAR-γδT cell approaches face challenges such as cytokine release syndrome (CRS), a potentially severe side effect caused by the massive release of cytokines (e.g., IL-6, TNF-α) from activated T cells or responding immune cells into the bloodstream [246,247]. This systemic inflammation requires careful monitoring and management strategies to ensure the safety and efficacy of these therapies in clinical settings. Additionally, the application of bispecific antibodies can facilitate direct and efficient interactions between γδT cells and tumor cells, significantly improving their anti-tumor functionality [248,249,250,251,252,253]. These advancements not only bolster current immunotherapies but also open new avenues for treating infectious diseases and autoimmune disorders.

Significant progress has also been made in integrating allogeneic γδT cells with conventional cancer treatments [141,254,255,256]. Studies indicate that combining γδT cells with chemotherapy, immune checkpoint inhibitors (such as anti-LAG3, PD1, or PDL1 antibodies), radiotherapy, or interventional therapy can effectively mitigate TME-induced immunosuppression, thereby enhancing anti-tumor activity. Notably, anti-LAG3 monoclonal antibody (mAb) therapy combined with γδT cell-based immunotherapy has been demonstrated to substantially augment immune responses [34]. However, under TME-induced stress, γδT cells may experience impaired chemotactic ability, preventing their effective migration to tumor sites [34]. In response, the development of novel bispecific antibodies presents a promising approach to improve γδT cell migration and tumor localization, thereby further enhancing therapeutic efficacy [252]. Additionally, localized administration of anti-BTN3A mAb [257] or zoledronic acid [258] within tumors may further expand the therapeutic potential of γδT cells. However, while such strategies enhance γδT cell efficacy, integrating them with immune checkpoint inhibitors like anti-PD-1 requires careful consideration of potential risks, particularly in specific HCC patient subsets. Recent research indicates that anti-PD-1 therapy may increase tumor growth in non-alcoholic steatohepatitis (NASH)-associated hepatocellular carcinoma (HCC) rather than reducing tumor burden [259]. In a mouse model, Pfister et al. (2021) found that anti-PD-1 treatment significantly promoted tumor growth in NASH-HCC, suggesting that in patients with underlying NASH, PD-1 inhibitors may pose a risk of disease progression. This highlights the importance of cautious patient selection and monitoring in this population. While our study focuses on γδT cell therapies, future investigations should explore whether these therapies carry similar risks or offer a safer alternative for patients with NASH-associated HCC.

The study of γδT cells has not advanced as rapidly as that of αβT cells, primarily because of the absence of specialized knockout mouse models. The difficulties stem from the complexity of γδT cell development, the limited understanding of its mechanism, and the influence of multiple genetic and signaling pathways on thymus development. In addition, because γδT cells lack clear markers and key transcription factors [143,144,260], the existing gene-editing technology makes it difficult to accurately knock out specific subsets. Compared with αβT cells, the number of γδT cells in the thymus is less, which makes the targeted research more difficult. At the same time, the complex interaction between γδT cells and thymus microenvironment may affect other T cell populations, which further increases the challenge of experimental design.

Despite many difficulties, researchers are actively promoting the establishment of γδT cell targeted gene knockout mouse model [261]. At the same time, the continuous optimization of the humanized mouse model also provides new possibilities for γδT cell research. For example, the use of human TCR transgenic mice combined with the transgenic expression of human BTN molecules is expected to further reveal the biological characteristics of γδT cells and lay the foundation for its application in immunological research.To summarize the current landscape of γδT cell-based cancer therapies and ongoing clinical trials, key findings and approaches are outlined in Table 2 below.

## 6. Conclusions

The heterogeneity and functional roles of γδT cells within the hepatic immune microenvironment are essential for immune surveillance, tumor immunity, and the regulation of liver diseases, including hepatitis, liver fibrosis, and hepatocellular carcinoma. As research advances, a deeper understanding of γδT cell subsets, their signaling pathways, and their interactions with other immune cells has elucidated their complex immunoregulatory functions across various pathological conditions. Despite significant progress in harnessing γδT cells for immunotherapy, several challenges persist, including immune evasion within the tumor microenvironment, cellular dysfunction, and limited migratory capacity. Future research efforts will be directed toward overcoming these limitations, particularly through engineering strategies designed to enhance anti-tumor efficacy, improve persistence, and optimize tumor targeting. These advancements hold great promise for developing more precise and effective therapies for liver diseases and cancer immunotherapy.

## Figures and Tables

**Table 1:** Subsets of Human γδT Cells and Their Functional Characteristics.

Vδ Chains	Paired Vγ Gene Usage	Distribution	Development in Thymus	Recognition/Antigen	Notes
Vδ1	Vγ2/3/4/5/8/9	Peripheral blood, skin, gut, spleen, liver	Mid-gestation onwards	MICA/B, ULBPs, B7-H6, CD1c, CD1d, SEB	Paired with diverse Vγ chains.
Vδ2	Vγ9	Peripheral blood	Detectable 5 to 6 weeks in fetal liver	Phosphoantigens, F1-ATPase, BTN3A1, hMSH2, MICA/B, SEs, TSST-1, Nectin-like 5	Vδ2/Vγ9 exclusively pairs.
Vδ3	Vγ2/3	Peripheral blood, liver	Predominant in late-fetal and neonatal blood	CD1d	Represent approximately 0.2% of the total circulating T cells and react to CD1d.
Vδ5	Vγ4	Peripheral blood	-	Endothelial protein C receptor (EPCR)	Identifying transformed cells by interacting with the endothelial protein C receptor.
Vδ6	/	Peripheral blood of lymphoma patients	-	-	-

Abbreviations: MICA/B, MHC Class I Chain-Related Proteins A and B; ULBP, UL16 Binding Protein; CD1c, Cluster of Differentiation 1c; CD1d, Cluster of Differentiation 1d; SEB, Staphylococcal Enterotoxin B; F1-ATPase, Mitochondrial ATP Synthase F1 Subunit; BTN3A1, Butyrophilin Subfamily 3 Member A1; hMSH2, Human MutS Homolog 2; TSST-1, Toxic Shock Syndrome Toxin-1.

**Table 2:** γδT Cell-Based Cancer Therapies and Clinical Trials.

Therapeutic Approach	Specific Strategy	Target Indications (Cancer Types)	NCT Number	Location
ACT (Adoptive Cell Transfer)	Donor/Allogeneic γδT Cell Infusion	Relapsed/Refractory Leukemia (e.g., AML)	NCT04439721	Suzhou, China
		Hepatocellular Carcinoma (HCC)	NCT04518774	Beijing, China
		Relapsed/Refractory NHL or PTCL	NCT04696705	Tianjin, China
		Relapsed Hematologic Malignancies	NCT05755854	Hefei, China
		Acute Myeloid Leukemia (AML)	NCT03790072	Praha, Czechia
	Autologous γδT Cell Infusion	Advanced HBV-Related Hepatocellular Carcinoma (HCC)	NCT04032392	Beijing, China
		Relapsed/Refractory B-NHL, CLL, PTCL	NCT04028440	Tianjin, China
	γδT Cells + Tumor Reduction Surgery (Cryoablation/IRE)	Hepatocellular Carcinoma (HCC)	NCT02425735,	Guangzhou, China
		Liver cancer		
			NCT03183219	
		Breast Cancer (Her-, Er-, Pr-)	NCT02418481, NCT03183206	Guangzhou, China
		Breast Cancer		
		Non-Small Cell Lung Cancer (No EGFR Mutation)	NCT02425748,	Guangzhou, China
		Lung cancer		
			NCT03183232	
		Locally Advanced Pancreatic Cancer	NCT03180437	Guangzhou, China
	γδT Cells + CIK	Gastric Cancer	NCT02585908	Beijing, China
CAR-γδT Cell Therapy	CD19-CAR-γδT Cells	B-Cell Lymphoma, ALL, CLL	NCT02656147	Beijing, China
		Relapsed/Refractory B-Cell NHL	NCT05554939, NCT06838832, NCT06503211	Beijing, China

				Nanjing, China
		Relapsed/Refractory B-Cell ALL	NCT06696833, NCT06056752	Suzhou, China Hefei, China
		Relapsed/Refractory B-Cell Hematologic Malignancies	NCT06092047	Suzhou, China
		B-Cell Malignancies (Follicular, Mantle Cell, Marginal Zone, Mediastinal)	NCT04735471	Stanford, CA, USA
	B7H3-CAR-γδT Cells	Advanced Solid Tumors	NCT06825455	Beijing, China
		Relapsed/Refractory B7H3+ Malignant Glioma	NCT06018363	Suzhou, China
		B7H3+ Solid Tumor Leptomeningeal Metastases	NCT06592092	Beijing, China
	GPC3/Mesothelin-CAR-γδT Cells	Solid Cancers (Expressing GPC3 or Mesothelin, e.g., HCC)	NCT06196294	Guangzhou, China
	Universal CAR-γδT Cells	Post-Transplant Relapsed AML	NCT04796441,	Yanda, China
		Refractory/Relapsed AML		
			NCT05388305	Wuhan, China
	CD7-CAR-γδT Cells	Relapsed/Refractory CD7+ T-Cell Malignancies	NCT04702841	Hefei, China
	UTAA17 (CAR-γδT Cells)	Relapsed/Refractory Multiple Myeloma (MM)	NCT06279026	Suzhou, China
	NKG2DL-CAR-γδT Cells	Relapsed/Refractory Solid Tumors (Colorectal, TNBC, Sarcoma, Nasopharyngeal)	NCT04107142	Johor, Malaysia
		Advanced Solid or Hematologic Malignancies	NCT05302037	Singapore
	CD70-CAR-γδT Cells	Relapsed/Refractory Clear Cell Renal Cell Carcinoma (ccRCC)	NCT06480565	Nashville, TN, USA
	Drug-Resistant γδT Cells (DRI)	Newly Diagnosed Glioblastoma (GBM)	NCT04165941	Birmingham, AL, USA
Combination Therapy	γδT Cells + Immunomodulators (Interferon/PD-1)	Stage III–IV Resectable Melanoma	NCT06212388	Xi’an, China
	TKC (NK + γδT Cells) + Chemotherapy	Advanced Non-Small Cell Lung Cancer (NSCLC)	NCT04990063	Xi’an, China

Note: All data in this table are sourced from https://clinicaltrials.gov/. Abbreviations: ACT (Adoptive Cell Transfer), CAR-γδT (Chimeric Antigen Receptor Gamma Delta T), NCT (National Clinical Trial), HCC (Hepatocellular Carcinoma), AML (Acute Myeloid Leukemia), NHL (Non-Hodgkin Lymphoma), PTCL (Peripheral T-Cell Lymphoma), B-NHL (B-Cell Non-Hodgkin Lymphoma), CLL (Chronic Lymphocytic Leukemia), ALL (Acute Lymphocytic Leukemia), MM (Multiple Myeloma), GBM (Glioblastoma Multiforme), TNBC (Triple-Negative Breast Cancer), ccRCC (Clear Cell Renal Cell Carcinoma), NSCLC (Non-Small Cell Lung Cancer), CIK (Cytokine-Induced Killer), IRE (Irreversible Electroporation).
